# Does Gender, Academic Status, Years of Teaching Experience, and Discipline Affiliation Affect Strategies Used to Promote Creativity in Medical Education at King Abdulaziz University?

**DOI:** 10.7759/cureus.36077

**Published:** 2023-03-13

**Authors:** Lana Shawwa, Mohammad K Yousef

**Affiliations:** 1 Medical Education Department, King Abdulaziz University, Jeddah, SAU; 2 Restorative Department, King Abdulaziz University Faculty of Dentistry, Jeddah, SAU

**Keywords:** gender, academic status, years of teaching experience, medical education, creativity

## Abstract

Background

This study aimed to explore the effect of gender, academic status, years of teaching experience, and discipline affiliation on strategies used to promote creativity in medical education.

Methodology

This study was conducted in the Faculty of Medicine, King Abdulaziz University, Jeddah, Saudi Arabia. Faculty teaching in both the basic sciences and clinical disciplines was included. A 15-item electronic survey was created. The frequencies were obtained for the demographic and academic characteristics of the participants. For each question (Q5-Q15), the mean and SD of participant responses were obtained. The chi-square test was used to compare all categorical variables. This included the comparison of the participants’ demographic characteristics with their academic characteristics. The chi-square test was also used for the comparison of participant characteristics among the categorical questions (Q5-Q15).

Results

A statistically significant association was determined between academic status, years of teaching experience, faculty’s perception of the curriculum, tasks used in teaching, and the suitability of the used assessment strategies in promoting creativity. Teaching in clinical disciplines was statistically associated with using real-life problems, challenging students, and deliberately creating chaos or disorder in the classroom.

Conclusions

Academic status, years of teaching experience, and discipline affiliation are more likely to affect how faculty members promote creativity in medical education.

## Introduction

Medical education is an essential part of the healthcare system. It not only prepares students for the health and well-being of their patients but also equips them to apply the principles of creative thinking in their daily interactions [1]. As healthcare systems are becoming progressively more complex, fast-changing, and unpredictable, healthcare providers must learn to be creative so that they can adapt appropriately and continue to perform effectively. Clinical presentations, contexts, and patient-care practices can shift from standard practices unexpectedly. Thus, equipping healthcare professionals with creative multidimensional perspectives is needed [2].

The relationship between creativity and medical education has gained attention in recent years, with studies highlighting its importance in shaping the skills and abilities of future medical professionals. Overall, incorporating creative activities into the medical education process can have a range of positive effects on students’ learning and development such as fostering critical thinking, problem-solving, and innovation, as well as improving scientific attitudes [2,3].

When looking at promoting creativity in medical education, one should start with the nature of the curriculum. For example, a curriculum that has interdisciplinary education elements where medical students collaborate with students from other health disciplines has been seen to encourage creative thinking and problem-solving [4].

A curriculum should also aim to incorporate diverse teaching strategies such as interactive lessons and games which increase students’ engagement along with the ability to teach important clinical and communication skills. The strategies should be primarily based on design-based learning, research-based learning, problem-solving, problem-based learning, project-based learning, and/or creative thinking [5,6]. Some studies have even specified that one might use activities such as drawing, writing, and music while others reported that storytelling can be an effective tool for teaching students about complex medical concepts and can also help students develop their communication and collaboration skills [7].

There are many other approaches that integrate creativity into the medical education system and the medical teacher remains the center of any attempt. In 2015, Cropley [8] reviewed the literature on creativity and summarized that the most effective teachers in promoting creativity are those who encourage independent learning, endure errors, take students’ questions seriously, and provide opportunities for students to work with diverse resources under diverse settings [8,9].

Having creative students is largely related to having a teacher with a creative mindset. Actions such as teaching process management, curriculum build-up, teaching strategies used, students’ assignments planned, and whether the teacher is a creative personality all play pivotal roles [9].

However, medical education can be a challenging field when it comes to promoting creativity and innovation. A major factor also revolves around faculty members and administration. Traditionally, individuals are inclined to resist change and instead favor maintaining the status quo and following routines [10]. Although most educators agree that developing creative learning approaches is important, not much has been done to promote creativity [11]. This may be due to focusing on teaching standards and competencies as well as the difficulty of teaching creativity and promoting new skills acquisitions [12,13]. Another reason is the belief that everything taught in education institutes must be measurable. This assessment using a standardized format might be hard, but it should not be a reason to stop promoting creativity in education curricula [14]. Several studies have shown that domain experts can assess creativity with an acceptable degree of reliability [15].

Other factors that might play a role in hindering creativity in medical education are the focus on memorization and fact-based learning [16], lack of time and resources, and numerous examinations and assessments to complete which can leave little time for creative thinking and exploration [17].

Although creativity is an important aspect of medical education, few studies have thoroughly explored faculty members’ creativity-supporting practices. Faculty members, as role models, can tremendously affect their students by sharing their passion for learning, empowering them to realize the potential and possibilities, stimulating students’ cognitive development, and encouraging their imagination skills [18].

It is critical to investigate how faculty members can foster such initiatives and create environments that support creativity in education. The global decline in creativity has been looming, and although significant progress in medical education in Saudi Arabia has been witnessed, studies of creativity in medical education have been scarce. The objective of this study is to correlate factors such as gender, academic status, years of teaching experience, and discipline affiliation with how faculty members are using creativity-promoting initiatives at the Faculty of Medicine (FOM), King Abdulaziz University (KAU). By understanding the characteristics that are associated with greater use of these initiatives, our educational institute can identify approaches to foster a culture of creativity and innovation among our faculty members.

## Materials and methods

Study design and setting

This was a cross-sectional study conducted by MED at KAU, FOM, Jeddah, Saudi Arabia, during the academic year 2022-2023.

Participants

All FOM faculty members teaching in basic medical sciences and clinical departments were included. The requirement of informed consent was waived by the Biomedical Ethics Research Committee at the FOM, KAU (reference number: 92-23 on 13-12-2022).

Description and data collection (survey)

A 15-item electronic survey was created. For initial validity evidence, we shared the questionnaire with statisticians and experts in medical education to evaluate the psychometric characteristics of the survey. The survey was also pretested on a smaller population of faculty (4%) of a total of 375 faculty members. For further validity, a pilot test was conducted on a subset of faculty members to evaluate the content and clarity of items.

The required sample was met for faculty members, and the response rate exceeded the expected proportion set in the sample size calculation where 211 faculty members responded. Furthermore, a post-hoc power of 0.97 or larger based on the smallest effect size (Cramer’s V = 0.220) in the study validated the sampling method at the desired margin of error.

To ensure maximum dissemination and participation of faculty members heads of departments were involved in distributing the anonymous survey using an official communication interface.

Statistical analysis

The quantitative data were entered into SPSS version 26.0 (IBM Corp., Armonk, NY, USA). The frequencies were obtained for the demographic and academic characteristics of participants. For each question (Q5-Q15) the mean and SD for the participant responses were obtained. The chi-square test was used for the comparison of all categorical variables. This included a comparison of participant demographic characteristics with their academic characteristics. The chi-square test was also used for the comparison of participant characteristics among the categorical questions (Q5-Q15).

## Results

Participants characteristics

The survey was sent to all 375 faculty members teaching in both basic sciences and clinical departments. A total of 213 faculty members responded. The descriptive statistics of participants are summarized in Table 1. The mean and SD for participant responses to creativity questions are shown in Table 2.

**Table 1:** Characteristics of participants.

Variable	Category	N = 213	%
Academic status	Professor	56	26.3%
	Associate Professor	56	26.3%
	Assistant Professor	80	37.6%
	Lecturer	21	9.9%
Gender	Male	110	51.6%
	Female	103	48.4%
Teaching experience	More than 15 years	97	45.5%
	10–14 years	32	15.0%
	5–9 years	39	18.3%
	Less than 5 years	45	21.1%
Teach in	Basic years	93	43.7%
	Clinical years	120	56.3%

**Table 2:** Mean and SD of participant responses to creativity questions.

Question	Mean and SD
There is sufficient time in the curriculum to allow my students to develop their creativity	3.05 (1.043)
Our curriculum allows my students to develop their creativity	3.02 (1.046)
When I use cases in my teaching, I use real-life problems to challenge my students	4.15 (0.874)
When teaching I deliberately create chaos or disorder in examples, questions, cases, and other situations to challenge students in the classroom	3.47 (1.127)
I give students assignments with enough time to work on and complete	3.34 (1.244)
In my course, I have tasks that enable creativity	3.08 (1.089)
I reserve time at the end of my teaching for questions and dialogue	4.38 (0.818)
My assessment strategies allow for answers that are not narrowly predetermined (fewer multiple-choice questions and more short essays)	2.54 (1.211)
In our department, there is a continuous academic debate and dialogue with various stakeholders about the role of creativity within our subjects	2.821 (1.227)
Besides lectures, I use diversified teaching strategies	3.61 (1.092)
I promote a psychologically safe environment where students are not afraid to express themselves	4.55 (0.729)

Gender and promoting creativity in medical education

Male faculty members were found to be significantly more likely to reserve time at the end of their teaching for questions and dialogue (p < 0.001). Gender did not have any effect on other approaches (Table 3).

**Table 3:** P-values of the four independent variables with the dependent variables.

Question	P-value between groups				
	Gender	Academic level	Experience in years	Teaching discipline basic or academic	
There is sufficient time in the curriculum to allow my students to develop their creativity	0.175	0.092	0.031	0.05	
					Our curriculum allows my students to develop their creativity	0.152	0.007	0.036	0.222	
										When I use cases in my teaching, I use real-life problems to challenge my students	0.759	0.379	0.045	<0.001>	
															When teaching, I deliberately create chaos or disorder in examples, questions, cases, and other situations to challenge students in the classroom	0.255	0.056	0.190	0.022	
																				I give students assignments with enough time to work on and complete	0.8	0.248	0.295	0.007	
In my course, I have tasks that enable creativity	0.124	0.032	0.220	0.048																					
					I reserve time at the end of my teaching for questions and dialogue	0.034	0.255	0.677	0.044																
										My assessment strategies allow for answers that are not narrowly predetermined (fewer multiple-choice questions and more short essays)	0.581	0.024	0.017	0.510											
															In our department, there is a continuous academic debate and dialogue with the various stakeholders about the role of creativity within our subjects	0.194	0.383	0.036	0.233						
																				Besides lectures, I use diversified teaching strategies	0.062	0.127	<0.001>	0.547	
I promote a psychologically safe environment where students are not afraid to express themselves	0.767	0.307	0.011	0.717																					

Academic status and promoting creativity in medical education

When evaluating if academic status influenced activities that promote creativity, it was found that professors and associate professors were significantly more likely to agree that the curriculum allows their students to develop their creativity (p < 0.001). In addition, they were found to be significantly more likely to agree that their courses have tasks that enable creativity (p < 0.001). The majority of faculty believed that the assessment strategies are narrowly pre-determined and do not allow for answers that show student thinking and analysis (p < 0.001) (Table 3).

Years of experience as a faculty member and promoting creativity in medical education

The faculty members who were employed and teaching for more than 15 years were found to be statistically more likely to believe that there is sufficient time in the curriculum to allow students to develop their creativity and that the nature of the curriculum allows students to develop their creativity (p < 0.001). When it came to teaching strategies, faculty members teaching for more than 15 years were found to be statistically more likely to use real-life problems to challenge their students (p < 0.001).

Faculty members working for nine years or less at KAU were statistically more likely to agree that the currently used assessment strategies are narrowly predetermined and do not allow students to show evidence of their thinking and problem-solving abilities (p < 0.001). They were also statistically more likely to agree that there is a continuous academic debate and dialogue with various stakeholders about the role of creativity within their subjects (p < 0.001).

Faculty members with 10 years and more of experience at KAU were statistically more likely to use diversified teaching strategies besides lectures (p < 0.001).

Faculty members with less than five years of experience at KAU were statistically more likely to agree that they are promoting a psychologically safe environment where students are not afraid to express themselves (p < 0.001) (Table 3).

Affiliation to disciplines and promoting creativity in medical education

When it came to faculty members teaching in basic years (second and third years), it was found that they were statistically more likely to give students assignments with enough time to work on and complete and that the tasks they assign enable creativity (p < 0.001).

Faculty teaching in clinical years (fourth, fifth, and sixth years) were statistically more likely to agree that there is sufficient time in the curriculum for students to develop their creativity (p < 0.001). When it came to teaching strategies, they were statistically more likely to use real-life problems to challenge their students, deliberately create chaos or disorder to challenge students in the classroom, and reserve time at the end of their teaching for questions and dialogue (p < 0.001) (Table 3).

## Discussion

Although the expected role of medical education is to accomplish the societal agreement for patient care, its responsibility has extended to include non-clinical skills such as leadership, innovation, and creativity [19]. Nowadays, despite key advances in medicine, the practice of medicine remains traditional and based on conventional principles. With the increasing prevalence of incurable diseases and the aging of the population, depending on traditional principles and ways of management is not enough. One must incorporate creative approaches that support the individual to respond in an appropriate way to challenges and difficulties [20]. Incorporating creativity in medical education not only empowers students to develop innovative solutions to complex clinical problems but also allows thinking critically and adapting to new daily challenges in the workplace. Knowledge alone is not enough, and it is essential to transform it to produce new ideas [21].

A central element to incorporating creativity in medical education is the faculty member. The role of faculty members extends beyond being knowledge teachers to serving as role models and mentors who share their own experiences and insights and provide guidance and support [22]. Teachers who support creativity are described as less rigid, less structured, more hands-on, and involve diverse ways of learning [23].

In this research, we were interested to explore how certain characteristics of our faculty members might affect creativity detrimental initiatives.

Years of experience and academic status

When we explored whether the curriculum is viewed as a vessel that enhances student engagement and creativity, we found that both years of teaching experience and academic status significantly affected the perception of the curriculum. Faculty members teaching for more than 15 years, whether they were professors or associate professors, were more likely to believe that there is sufficient time in the curriculum to allow students to develop their creativity and that the nature of the curriculum allows students to develop their creativity. Exploring this finding, one must address the faculty involvement in curriculum committees. Those faculty members are either fully involved in the curriculum committee, can see the bigger picture, and are able to clearly realize where creativity is embedded in the curriculum, or they are not and are judging based on their limited discipline experience. Hence, more exploration of their perception is needed along with involvement in the curriculum development committee to incorporate creativity [24].

When it came to teaching strategies, faculty members with 15 years of teaching experience were more likely to use real-life problems to challenge their students. Several studies have reported that the use of real-life problems promotes active learning by emphasizing the aspects of analysis, synthesis, and critical reasoning, as well as high-level cognitive skills that can foster creative thinking [25,26].

The years of experience might have been helpful in increasing the faculty’s ability to develop more effective teaching methods and strategies. This can be explained when understanding the effect of time and experience where faculty members with time will have a deeper understanding of the subject matter and will be able to provide more diverse and nuanced perspectives, leading to a more well-rounded education. This can also be explained by prolonged exposure to the changing faculty development culture at FOM where programs and workshops might have acted as a trigger to use creativity-supportive approaches. Exploring the reasons for using such approaches in depth might direct us to a genre of faculty who are more inclined to have improvement-focused learning goals, appreciate creative work, and believe that their teaching is not simplistic [27].

Although more years of teaching experience have been seen to have a positive effect, one must also be mindful that faculty members with more experience may become more set in their ways and less receptive to new and innovative ideas, which can limit the opportunities for creativity. Moreover, the longer the experience the higher the tendency to rely on traditional teaching methods and materials, which may not be as effective in fostering creativity as newer, more innovative approaches [28].

Ultimately, we believe that the effect of years of experience on promoting creativity in medical education will depend on the individual faculty member’s mindset, teaching style, and willingness to adapt to new ideas and methods [29]. Faculty members who actively seek out new and innovative approaches and encourage student creativity are likely to be more effective in promoting creativity, regardless of their years of experience [30].

Faculty members with higher academic status may have a greater depth of knowledge in their medical education field, which can provide a rich foundation for promoting creative thinking in their students. They may also have access to more resources and opportunities for research and innovation, which can inspire and motivate students to pursue their own creative projects [31]. However, it is important to note that academic status alone does not necessarily equate to a greater ability to promote creativity. Faculty members with lower academic status can still be effective in promoting creativity if they are committed to creating a supportive and stimulating learning environment that encourages students to think outside the box.

Discipline affiliation and creativity

Looking at discipline affiliation, faculty teaching in clinical years is more likely to agree that there is sufficient time in the curriculum for students to develop their creativity, use real-life problems to challenge their students, deliberately create chaos or disorder to challenge students in the classroom, and reserve time at the end of their teaching for questions and dialogue. This can be explained by the extensive experience gained in the clinical setting, which can provide a unique perspective on the challenges and opportunities associated with delivering high-quality patient care. Clinical educators may also have access to cutting-edge technologies and treatment approaches that can inspire and motivate students to explore new and innovative ideas. It is very important to empower clinical medical faculty members because they are also uniquely positioned to help students bridge the gap between theory and practice and inspire students to think critically and creatively about the challenges and opportunities associated with delivering high-quality patient care [32].

On exploring the effect of teaching in basic sciences, we found that they are more likely to give students assignments with enough time to work on and complete and that the tasks they assign enable creativity. One can propose that faculty teaching in basic sciences have a deep understanding of the fundamental principles that underlie medical practice, which can provide a rich foundation for promoting creative thinking in their students. They can promote creativity in medical education by encouraging students to think critically about basic science concepts and how they apply to clinical practice, as well as by providing opportunities for students to explore new and innovative research projects and ideas [33].

Creativity and assessment strategies at the Faculty of Medicine

The majority of faculty, in general, and especially the faculty who have been teaching for fewer than nine years agreed that the assessment strategies used at FOM are narrowly predetermined and do not allow for answers that illustrate student thinking and analysis.

In his review, Lucas [34] found multiple assessment instruments for the development of specific traits linked to creativity, including the use of descriptive rubrics supported by examples, assessment by peers, assessment using portfolios, assessment using mixed methods, and various forms of self-assessment.

One of the essential characteristics of creativity is the ability of students to show divergent thinking, which is the ability to generate multiple solutions [34]. With conventional standardized tests, students are encouraged to share the right answer and avoid the wrong ones [35]. For example, this is evident with multiple-choice or short-answer questions that require one right answer. Problems that require divergent thinking are unintentionally devalued using standardized tests. Proposing using essay-type tests might be effective in presenting divergent thinking unless they are scored against one or more implicit prototypes or model answers which will discourage creativity [36].

Promoting creativity means epitomizing a balance between knowledge and freeing oneself of that knowledge [37]. Knowledge is essential but might hinder creativity [36]. The challenge is that although in our research the faculty might be using initiatives to promote creativity in the classroom when it comes to assessment strategies, knowledge is what they seek. The question here is how we can align the use of creativity-promoting teaching activities with assessment activities that show evidence of student creativity. The first step might be through raising awareness among faculty members that creativity is not one solid entity but rather constitutes a confluence of elements.

In 2016, Lucas [34] introduced the five-dimensional model of creativity. The model has five core creative habits, namely, inquisitive, imaginative, persistent, collaborative, and disciplined. Sternberg proposed that creativity includes six different but interconnected elements, namely, intellectual abilities, knowledge, styles of thinking, personality, motivation, and environment. Irrespective of the model used to explore creative elements, one must be mindful that there is a certain threshold for each element and that interactions among these elements are existent. In conclusion, realizing that creativity involves a system of categories should be the basis when creating assessment strategies [38].

In 2013, Brookhart [38] suggested a rubric that might be helpful for both faculty members and students by acting as a visual reminder that makes creativity stand out from other assessment criteria. The rubric describes four levels of creativity in four different areas such as the variety of ideas, the variety of sources, the novelty of idea combinations, and the novelty of communication. In his work, Brookhart stressed that the rubric should not be used as a grading instrument but rather to clarify criteria for success and show what the continuum of performance looks like [38].

Because creativity is a broad concept, no single approach is effective in addressing it. The medical education department should start to initiate and support academic debates about creativity using rubrics, examples, and faculty development programs.

Study limitations

While this study looked at faculty members’ characteristics that affect their choice of creativity-promoting initiatives, we believe that it is imperative to understand from students’ perspectives if these initiatives are in fact being implemented. Involving students by asking them to validate these results is vital in understanding the efficacy of these initiatives. Although faculty members did not receive formal training on creativity, it would have been informative to explore from where faculty members gathered their knowledge regarding creativity in medical education. Furthermore, we must also validate findings related to the assessment strategies used at FOM with the assessment unit administrators to ascertain whether these assessment strategies hinder the act of assessing creativity in medical education.

## Conclusions

Characteristics such as gender, years of teaching experience, academic status, and discipline affiliation are likely to affect how faculty members promote creativity in medical education and their engagement and use of creativity-promoting initiatives. Understanding the effect of such characteristics can help the medical education department design and implement more effective programs that are tailored to the needs and preferences of their faculty members. Furthermore, these findings will help better target efforts and resources toward faculty members who are most likely to benefit from them. To ensure the maximum benefits one might start by creating a task force of faculty members that will aid in promoting a culture of incorporating creativity in medical education. Initiatives and achievements should be highlighted, shared, and rewarded. Involving junior faculty members in a proper mentoring program and considering innovation as an academic culture might also be helpful. Finally, identifying characteristics can also help to develop targeted professional development programs that address the specific needs and interests of different groups of faculty members. This can lead to more effective training and support programs and ultimately contribute to the overall success of our institution.

## References

[1] Zubaidah S, Fuad N, Mahanal S, Suarsini E (2017). Improving creative thinking skills of students through differentiated science inquiry integrated with mind map. J Turkish Sci Educ.

[2] Ten Haven A, Pragt E, Luijk SJ, Dolmans DH, van Mook WN (2022). Creativity: a viable and valuable competency in medicine? A qualitative exploratory study. Med Teach.

[3] Duran M (2016). The effect of the inquiry-based learning approach on student's critical thinking skills. Eurasia J Math Sci Technol Educ.

[4] Kuo HC, Tseng YC, Yang YT (2019). Promoting college student’s learning motivation and creativity through a STEM interdisciplinary PBL human-computer interaction system design and development course. Thinking Skills Creativity.

[5] Seechaliao T (2017). Instructional strategies to support creativity and innovation in education. J Educ Learn.

[6] Iyengar E, Meier P, Hamelers R (2017). The small mammal project: engaging students as scientists. Am Biol Teacher.

[7] Catala A, Theune M, Gijlers H, Heylen D (2017). Storytelling as a creative activity in the classroom. Proceedings of the 2017 ACM SIGCHI Conference on Creativity and Cognition.

[8] Cropley D (2015). Through the looking glass: inside the world of creativity research. Creativity Res J.

[9] Mukan N, Yaremko H, Kozlovskiy Y, Ortynskiy V, Isayeva O (2019). Teachers' continuous professional development: Australian experience. Adv Educ.

[10] Hon AH, Bloom M, Crant JM (2011). Overcoming resistance to change and enhancing creative performance. J Manage.

[11] Plucker JA (2022). The patient is thriving! Current issues, recent advances, and future directions in creativity assessment [in press]. Creativity Res J.

[12] Baer J, Garrett T (2012). Teaching for creativity in an era of content standards and accountability. Nurturing Creativity in the Classroom.

[13] Baer J (2002). Are creativity and content standards allies or enemies. Res School.

[14] Baer J (2015). Domain Specificity of Creativity. Academic Press.

[15] Amabile TM (1997). Motivating creativity in organizations: on doing what you love and loving what you do. Calif Manage Rev.

[16] Nasrollahi-Mouziraji A, Nasrollahi-Mouziraji A (2015). Memorization makes progress. Theory Pract Lang Stud.

[17] Alencar EM, Fleith DS, Pereira N (2017). Creativity in higher education: challenges and facilitating factors. Temas em Psicologia.

[18] Crane R (2017). Mindfulness-Based Cognitive Therapy: Distinctive Features. https://www.taylorfrancis.com/books/mono/10.4324/9781315627229/mindfulness-based-cognitive-therapy-rebecca-crane.

[19] O'Connor E (2019). Cognitive apprenticeship in the ICU: ward round activities to enhance student learning. Med Teach.

[20] Csikszentmihalyi M (2014). The systems model of creativity and its applications. The Wiley Handbook of Genius.

[21] Corazza GE (2016). Potential originality and effectiveness: the dynamic definition of creativity. Creativity Res J.

[22] Sidek R, Halim L, Buang NA (2022). Pedagogical approaches to inculcate scientific creativity among secondary students. Creative Educ.

[23] Dalke AF, Cassidy K, Grobstein P, Blank D (2007). Emergent pedagogy: learning to enjoy the uncontrollable—and make it productive. J Educ Change.

[24] Alsubaie MA (2016). Curriculum development: teacher involvement in curriculum development. J Educ Pract.

[25] DeHaan RL (2011). Science education. Teaching creative science thinking. Science.

[26] Greenstein LM (2012). Assessing 21st Century Skills. A Guide to Evaluating Mastery and Authentic Learning. Corwin Press.

[27] Beghetto RA (2013). Killing Ideas Softly?: The Promise and Perils of Creativity in the Classroom. IAP.

[28] Buehl MM, Beck JS (2014). The relationship between teachers' beliefs and teachers' practices. International Handbook of Research on Teachers' Beliefs.

[29] Trust T, Krutka DG, Carpenter JP (2016). “Together we are better”: professional learning networks for teachers. Computers Educ.

[30] Ferrari A, Cachia R, Punie Y (2009). Innovation and Creativity in Education and Training in the EU Member States: Fostering Creative Learning and Supporting Innovative Teaching. JRC Technical Note.

[31] Acar OA, Tarakci M, Van Knippenberg D (2019). Creativity and innovation under constraints: a cross-disciplinary integrative review. J Manage.

[32] Yendol-Hoppey D, Dana NF, Hoppey DT (2019). Preparing the Next Generation of Teacher Educators for Clinical Practice. IAP.

[33] Kulasegaram KM, Martimianakis MA, Mylopoulos M, Whitehead CR, Woods NN (2013). Cognition before curriculum: rethinking the integration of basic science and clinical learning. Acad Med.

[34] Lucas B (2016). A five-dimensional model of creativity and its assessment in schools. Appl Measure Educ.

[35] Gardner H (2006). Assessment in context: the alternative to standardized testing. The Development and Education of the Mind.

[36] Sternberg RJ (2012). The assessment of creativity: an investment-based approach. Creativity Res J.

[37] Johnson-Laird PN (1988). Freedom and constraint in creativity. The Nature of Creativity: Contemporary Psychological Perspectives.

[38] Brookhart SM (2013). Assessing creativity. Educ Leadership.

